# Cantú syndrome with coexisting familial pituitary adenoma

**DOI:** 10.1007/s12020-017-1497-9

**Published:** 2018-01-11

**Authors:** Pedro Marques, Rupert Spencer, Patrick J. Morrison, Ian M. Carr, Mary N. Dang, David T. Bonthron, Steven Hunter, Márta Korbonits

**Affiliations:** 1 Centre for Endocrinology, William Harvey Research Institute, Barts and the London School of Medicine and Dentistry, Queen Mary University of London, London, UK; 2 Department of Medical Genetics, Belfast HSC Trust, Belfast, UK; 3 School of Medicine, St James’s University Hospital, University of Leeds, Leeds, UK; 4 Regional Centre for Endocrinology and Diabetes, Royal Victoria Hospital, Belfast, UK

**Keywords:** Cantú syndrome, ABCC9, pseudoacromegaly, familiar pituitary adenoma

## Abstract

**Context:**

Pseudoacromegaly describes conditions with an acromegaly related physical appearance without abnormalities in the growth hormone (GH) axis. Acromegaloid facies, together with hypertrichosis, are typical manifestations of Cantú syndrome.

**Case description:**

We present a three-generation family with 5 affected members, with marked acromegaloid facies and prominent hypertrichosis, due to a novel missense variant in the *ABCC9* gene. The proband, a 2-year-old girl, was referred due to marked hypertrichosis, noticed soon after birth, associated with coarsening of her facial appearance. Her endocrine assessment, including of the GH axis, was normal. The proband's father, paternal aunt, and half-sibling were referred to the Endocrine department for exclusion of acromegaly. Although the GH axis was normal in all, two subjects had clinically non-functioning pituitary macroadenomas, a feature which has not previously been associated with Cantú syndrome.

**Conclusions:**

Activating mutations in the *ABCC9* and, less commonly, *KCNJ8* genes—representing the two subunits of the ATP-sensitive potassium channel—have been linked with Cantú syndrome. Interestingly, minoxidil, a well-known ATP-sensitive potassium channel agonist, can cause a similar phenotype. There is no clear explanation why activating this channel would lead to acromegaloid features or hypertrichosis. This report raises awareness for this complex condition, especially for adult or pediatric endocrinologists who might see these patients referred for evaluation of acromegaloid features or hirsutism. The link between Cantú syndrome and pituitary adenomas is currently unclear.

## Introduction

The term pseudoacromegaly is used to describe cases where an acromegaly related physical appearance can be observed without any abnormality in the growth hormone (GH) axis. Coarse facial appearance with hypertrichosis are typical manifestations of Cantú syndrome [1–3].

Cantú syndrome, also known as hypertrichotic osteochondrodysplasia, is a heterogeneous condition that usually includes acromegaloid facial features, hypertrichosis, as well as skeletal and cardiac abnormalities (Table 1) [1, 4, 5]. Earlier reports have used different terms such as acromegaloid facial appearance (AFA) syndrome [6] or hypertrichosis acromegaloid facial features (HAFF) syndrome following the report of a family with 4 members affected with an AFA and congenital generalized hypertrichosis [2]. These conditions are phenotypically overlapping with Cantú syndrome and in fact represent a spectrum of the same condition. Following the description of activating *ABCC9* mutations in Cantú syndrome [1, 5], we have analyzed a family published 20 years ago by Irvine [2] and identified a novel missense *ABCC9* variant carried by the affected members.

**Table 1:** Major clinical features of Cantú syndrome

Cantú syndrome clinical manifestations	III.3	II.8	II.3	III.1	Molecularly proven Cantú syndrome *n*** = **30 [1, 4, 5, 15, 16**]**
Cranio-facial dysmorphology					
Coarse facial appearance	+	+	+	+	30/30 [1, 4, 5, 15, 16]
Broad nasal bridge	+	+	+	+	24/26 [1, 5, 15]
Bulbous nose	+	+	+	+	29/30 [1, 4, 5, 15, 16]
Small nose/anteverted nostrils	**−**	**−**	−		11/13 [5, 15]
Prominent mouth with thick lips	+	+	+	**−**	29/30 [1, 4, 5, 15, 16]
Long philtrum	+	+	+	+	28/29 [1, 4, 5, 15]
Macroglossia	+	+	+	+	15/28 [1, 5, 15, 16]
Gingival hyperplasia	**−**	**−**	**−**	+	10/18 [1, 4, 5, 16]
High or narrow palate	**−**	**−**	**−**	**−**	9/12 [5, 16]
Anterior open bite	**−**	**−**	**−**	**−**	3/11 [5]
Epicanthal folds	**−**	**−**	**−**	**−**	19/27 [1, 5, 15]
Short neck	**−**	**−**	**−**	**−**	5/11 [5]
Multiple labial frenula	**−**	**−**	**−**	**−**	One single case [16]
Hair					
Congenital generalized hypertrichosis	+	+	+	+	30/30 [1, 4, 5, 15, 16]
Abundant/curly eyelashes	**−**	**−**	**−**	**−**	9/11 [5]
Spiky hair	**−**	**−**	**−**	**−**	2/14 [1]
Cardiovascular					
Cardiomegaly	**−**	**−**	+	**−**	15/30 [1, 5, 15, 16]
Concentric hypertrophy of the ventricles	**−**	**−**	**−**	**−**	13/30 [1, 4, 5, 15, 16]
Pericardial effusion	**−**	+	**−**	**−**	4/29 [1, 4, 5, 15]
Pulmonary hypertension	n.a.	n.a.	n.a.	n.a.	4/29 [1, 4, 5, 15]
Patent ductus arteriosus	n.a.	n.a.	n.a.	n.a.	11/16 [1, 4]
Patent foramen ovale	n.a.	n.a.	n.a.	n.a.	2/16 [1, 15]
Atrial septal defects	**−**	**−**	**−**	**−**	2/14 [1]
AV block or fascicular block	**−**	**−**	**−**	**−**	1/2 [15]
Thoracic aorta aneurism	n.a.	n.a.	n.a.	n.a.	One single case [15]
Myocarditis	**−**	**−**	**−**	**+**	
Skeletal abnormalities					
Thickened calvarium	**−**	**−**	+	**−**	9/30 [1, 4, 5, 15, 16]
Craniosynostosis	**−**	**−**	**−**	**−**	1/2 [15]
Broad ribs	**−**	**−**	**−**	**−**	16/30 [1, 4, 5, 15, 16]
Narrow thorax	**−**	**−**	**−**	**−**	4/11 [5]
Platyspondyly and ovoid vertebral bodies	n.a.	n.a.	n.a.	n.a.	5/26 [1, 5, 16]
Narrow obturator foramen	n.a.	n.a.	n.a.	n.a.	2/11 [5]
Coxa vara/valga	**−**	**−**	**−**	**−**	3/11 [5]
Scoliosis	**−**	**−**	**−**	**−**	6/27 [1, 4, 5]
Osteopenia	n.a.	n.a.	n.a.	n.a.	2/12 [5, 16]
Delayed bone age	−	n.a.	n.a.	n.a.	3/12 [5, 16]
Hypoplastic bones	**−**	**−**	**−**	**−**	2/26 [1, 5, 16]
Erlenmeyer flask-like long bones with metaphyseal flaring	n.a.	n.a.	n.a.	n.a.	6/26 [1, 5, 16]
Hyperextensibility of joints	**−**	**−**	**−**	**−**	15/27 [1, 4, 5]
Enlarged medullary canal	n.a.	n.a.	n.a.	n.a.	8/12 [5, 16]
Pectus carinatum	**−**	**−**	**−**	**−**	2/11 [5]
Skin					
Loose, soft and/or wrinkled skin	−	−	−	−	18/27 [1, 5, 15]
Deep palmar and plantar creases	−	−	−	−	14/27 [1, 5, 15]
Persistent fingertip pads	−	−	−	−	12/26 [1, 5, 16]
Keloid formation	−	−	−	−	One single case [16]
Endocrine system					
Enlarged pituitary sella turcica	−	−	−	−	One single case [5]
Pituitary hyperplasia	−	−	−	−	One case with CS phenotype, not proven molecularly [11]
GH deficiency	−	−	−	−	One single case associated to *KCNJ8* gene mutation [8]
Pituitary adenoma	+	−	+	−	No reported cases
Other manifestations					
Macrosomia at birth (adult height usually normal)	−	−	−	−	19/29 [1, 4, 5, 15]
Polyhydramnios	n.a.	n.a.	n.a.	n.a.	12/29 [1, 4, 5, 15]
Developmental and/or speech delay	−	−	−	−	10/29 [1, 4, 5, 15]
Edema/ lymphedema	−	−	−	−	5/11 [5]
Pyloric stenosis	−	−	−	−	1/11 [5]
Feeding problems and poor intestinal motility	−	−	−	−	8/14 [1]
Hepatomegaly/ splenomegaly	−	−	−	−	2/14 [1]
Immune dysfunction and recurrent infections	−	−	−	−	11/27 [1, 5, 15]
Tracheo/broncho/laryngomalacia	n.a.	n.a.	n.a.	n.a.	3/14 [1]
Hoarse voice	−	−	−	−	3/14 [1]
Large hands	−	−	+	−	2/3 [15, 16]
Umbilical hernia	−	−	−	−	5/12 [5, 15]
Renal abnormalities	−	−	−	−	1/11 [5]
Genital abnormalities	−	−	+ (small uterus)	−	3/12 [5, 16]
Neurological manifestations					
Migraines	**−**	−	−	−	5/10 [12]
Seizures	**−**	−	−	−	2/10 [12]
Hypotonia	**−**	−	−	−	3/10 [12]
Autism	**−**	−	−	−	1/10 [12]
Attention difficulties and behavioral problems	**−**	−	−	−	4/10 [12]
Cerebral atrophy	**−**	−	−	−	2/10 [12]
White matter changes	+	−	−	−	3/10 [12]
Tortuous cerebral vasculature	**−**	−	−	−	5/10 [12]
Tortuous retinal vessels	n.a.	n.a.	n.a.	n.a.	2/10 [12]

Features present in the reported alive family members are marked with (+); absent features are marked with (−); features that are unknown or were not actively investigated are marked with (n.a.). The right column shows the presence of manifestations in patients with mutation positive Cantú syndrome reported in the literature

We aim to raise awareness of this complex condition, with prominent features resembling endocrine conditions and having significant cardiological complications. Moreover, we highlight a potential link between familial pituitary adenomas and Cantú syndrome.

## Case description

**Fig. 1:**
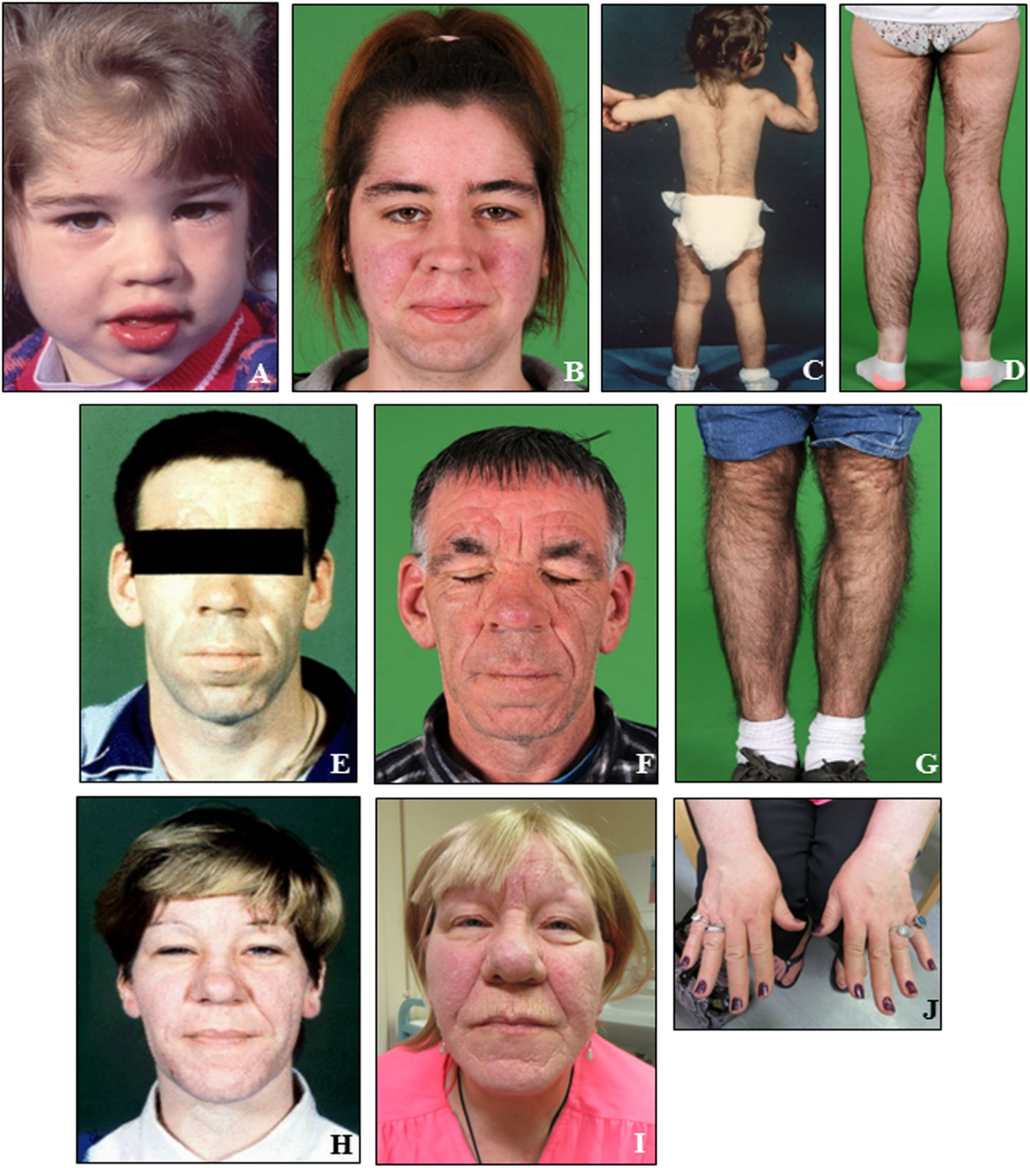
Facial appearance and generalized terminal hypertrichosis of the proband at the ages of 2 (**a**, **c**) and 22 years (**b**, **d**). The proband's father at the ages of 28 (**e**) and 48 years (**f**, **g**), and the proband's paternal aunt at the ages of 36 (**h**) and 57 years (**i**, **j**)

**Fig. 2:**
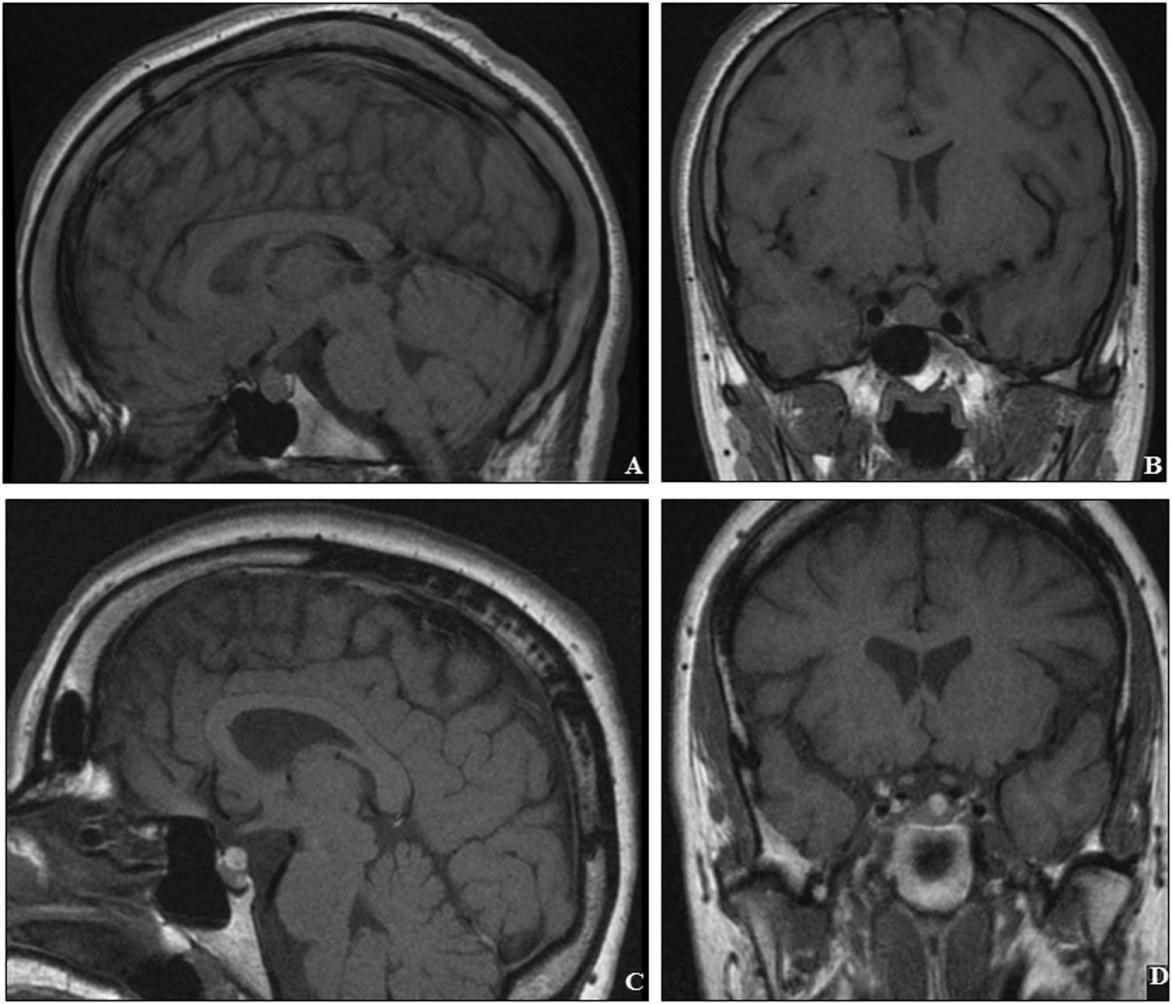
Pituitary imaging investigations in the proband (**a**, **b**) and the proband's paternal aunt (**c**, **d**). Mildly thickened calvarium can be seen (**c**)

The proband (III.3) was referred at age of 2 years to the Dermatology department due to prominent generalized hypertrichosis, noticed soon after birth, and coarsening facial appearance, with broadening of her nose and lower lip thickening (Fig. 1a–d). Her height and weight were just below the 97th centile, with her bone age matching the chronological age. Baseline pituitary function assessment was normal, including the GH axis. Over the following 20 years, her acromegaloid features and hypertrichosis progressed (Fig. 1b, d). The patient manages her hypertrichosis cosmetically and with clothing. Her final adult height is 171 cm (above the 90th centile). At the age of 14 years she was diagnosed with a 12 mm non-functioning pituitary adenoma (Fig. 2), which has been stable in size over the last 8 years.

The proband's father (II.8) was referred to the Endocrinology department due to a clinical suspicion of acromegaly, particularly because of acromegaloid facies (Fig. 1e). His GH axis and pituitary MRI scan were normal. Over the last 20 years, his acromegaloid features have been stable (Fig. 1f, g). At the age of 24 years he presented with non-specific chest pain and shortness of breath and was found to have a pericardial effusion for which no cause was identified. He later had repeated pericardiocentesis for recurrent effusions and subsequently had pericardial fenestration at the age of 30 years.

The proband's paternal aunt (II.3) was first seen at the Endocrinology department for exclusion of acromegaly. In addition to her acromegaloid facial appearance (Fig. 1h), she had terminal hypertrichosis. Her GH axis assessment was normal, with a normal serum IGF-1. Twenty years later, progression of coarse facial features is noticeable (Fig. 1i–j), while the hypertrichosis has remained stable requiring no specific treatment. At age of 44 years she was diagnosed with a 13 mm non-functioning pituitary adenoma (Fig. 2), unchanged in size over the last 14 years. She was noted to have mild hyperprolactinemia, likely due to a stalk effect (1030 mU/l [NR < 500]), and secondary adrenal insufficiency was also documented (suboptimal cortisol peak of 461 nmol/l on an insulin tolerance test, and 300 nmol/l on a short *Synacthen* test) for which she was commenced on hydrocortisone replacement therapy. Moderate thickening of the posterior calvarium was identified on a skull X-ray, and also noted on the MRI images (Fig. 2c). She was noted to have cardiomegaly, although she does not have hypertension or valve abnormalities. She has been recently diagnosed with a grade III infiltrating ductal breast carcinoma; one of her 53-year-old sisters had the same condition. *BRCA1* and *BRCA2* genetic testing did not reveal any abnormality.

The proband's half-sister (III.1) was referred to the Endocrinology department due to coarse facial features, a prominent forehead, thickened lips, long philtrum, and enlarged nose, and hypertrichosis. Her endocrine assessment was normal, including a normal serum IGF-1 and normal pituitary CT scan. At the age of 25 years she had an episode of chest pain associated with a mild troponin elevation, with a 15% rise on a second sample, attributed to a myocarditis.

The proband's grandfather (I.2), described as “hairy”, was never assessed by the genetic or medical departments.

**Fig. 3:**
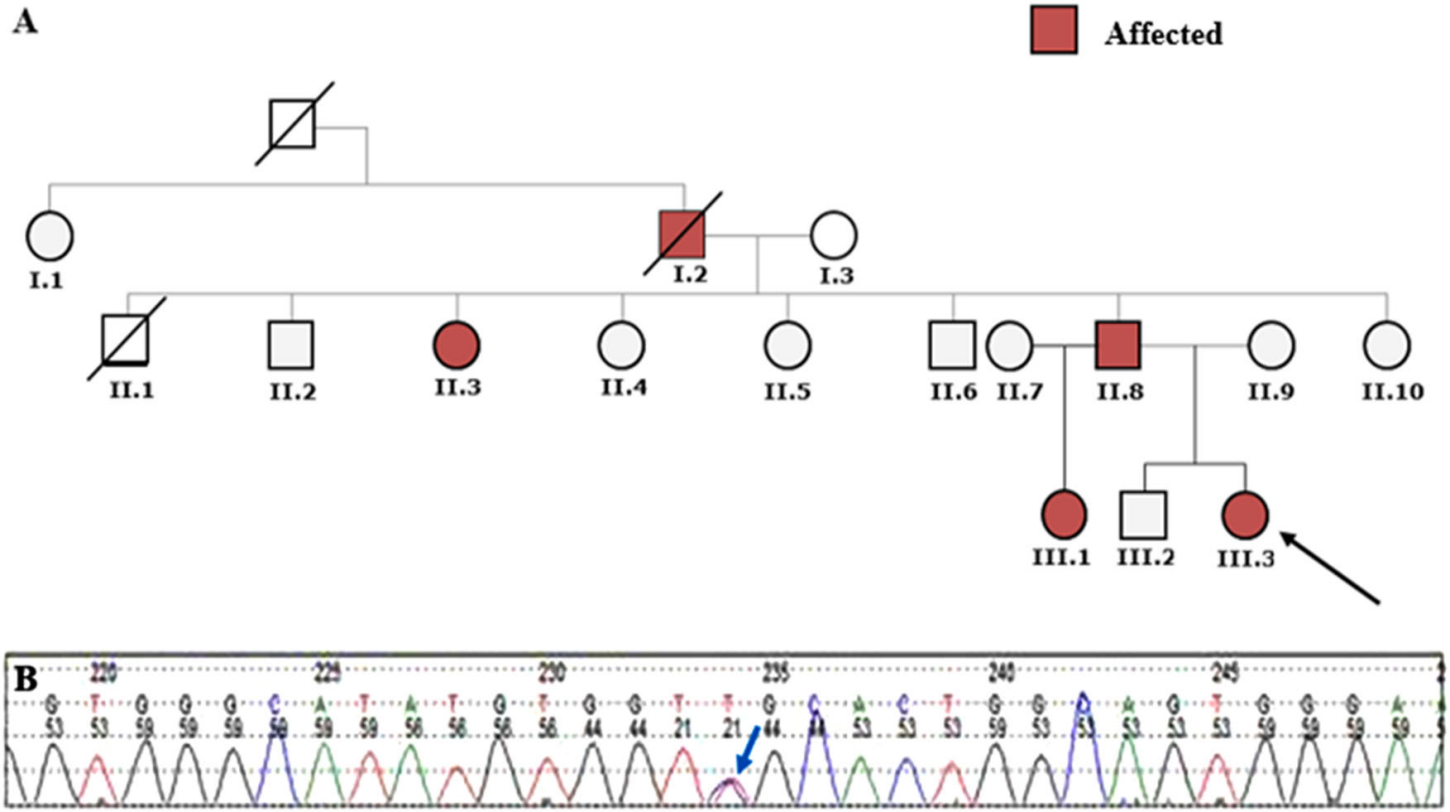
**a** Pedigree tree of our kindred; the proband, subject III.3, is marked with an arrow. **b** Sequencing electropherogram of the proband’s DNA. The double peak (blue arrow) showing a novel heterozygous missense variant at c.4039 C > T (p.Arg1347Cys)

The pedigree is consistent with an autosomal dominant inheritance pattern (Fig. 3) [2].

## Genetic testing

The *ABCC9* gene has been linked with Cantú syndrome in 2012 [1, 5], and some of the patients previously described as suffering from AFA and HAFF syndromes, were also identified with mutations in *ABCC9* [4]. *ABCC9* encodes a member of the superfamily of adenosine triphosphate (ATP)-binding cassette transporter subfamily C, commonly referred to as SUR2 (sulfonylurea receptor 2) protein. This transmembrane protein functions as a subunit of ATP-sensitive potassium channels in cardiac, skeletal, vascular, and non-vascular smooth muscle, and other tissues. Co-expression of SUR2 with the pore-forming inward rectifier proteins, Kir6.1 (encoded by *KCNJ8*) or Kir6.2 (*KCNJ11*) generates functional ATP-sensitive potassium channels [3]. All pathogenic variants in *ABCC9* reported to date in Cantú syndrome are gain-of-function missense mutations [1, 3, 5]. Activation of *ABCC9* reduces ATP-mediated potassium channel inhibition, thereby opening the channel [1, 5]. More rarely, Cantú syndrome can be caused by mutations in the *KCNJ8* gene [7].

We sequenced the *ABCC9* gene and identified a novel missense variant in the affected subjects: c.4039 C > T (p.Arg1347Cys) (Fig. 3). This missense variant, not reported in the literature and not present in the GnomAD database, causes a substitution of a highly conserved arginine residue for a cysteine at codon 1347 in the second nucleotide binding domain of *ABCC9*. In silico bioinformatics analysis (SIFT and PolyPhen) supports the pathogenicity of this variant.

## Discussion

The prevalence of Cantú syndrome is unknown. Males and females are equally affected and there is no established phenotype–genotype correlation. This conditions is inherited in an autosomal dominant manner, and penetrance thus far appears to be complete [3, 8].

It is currently unclear as to how activating *ABCC9* mutations lead to hypertrichosis, acromegaloid facial features, osteochondrodysplasia, and cardiovascular anomalies, while these features remarkably overlap with the side-effects of minoxidil, which binds to SUR2 resulting in ATP-sensitive potassium channel opening and activation [3]. Minoxidil promotes keratinocyte proliferation, glycosaminoglycan, and elastin production from skin fibroblasts, thereby changing connective tissue composition [9]. Regarding hypertrichosis, potassium channel opening, with consequent vasodilatation, may increase the blood supply, oxygen, and nutrients to the hair follicles leading to hair growth. Cardiovascular effects have been attributed to reduced vascular tone, which may explain pericardial effusions seen in Cantú syndrome patients [3, 10] and minoxidil-treated patients [10]. ATP-sensitive potassium channels are expressed in chondrocytes and osteoblasts, but their role in bone maturation as the explanation for skeletal abnormalities in *ABCC9*-related disorders is unknown [3].

No major endocrinopathies have been reported in Cantú syndrome [11]. The GH axis, often investigated due to possible acromegaly (the main differential diagnostic entity), has been shown to be normal [1, 3–5]. There is, however, one single case of a boy with Cantú syndrome due to a *KCNJ8* gene mutation found with GH deficiency [7]. No pituitary adenomas have been reported in Cantú syndrome, despite the fact that these patients commonly undergo brain imaging as part of investigations for neurological symptoms or as a routine procedure to exclude cerebrovascular abnormalities (Table 1) [4]. No pituitary adenomas were reported in a series of ten patients with genetically confirmed Cantú syndrome who had neuroimaging studies [12]. Scurr et al. reported one patient with a mild pituitary fossa enlargement and a moderate enlargement of the pituitary gland (10 × 11 mm) extending into the suprasellar cistern, but no pituitary adenoma was visible in this case [11]. In our kindred, we have two cases with non-functioning pituitary adenoma. Although pituitary adenomas are not rare in the general population, most are small incidentally found lesions [13]. Here we report pituitary macroadenomas in two family members, one found at the age of 14 years. These may represent a Cantú syndrome-related feature or the independent disease of familial isolated pituitary adenoma [14].

The differential diagnosis for Cantú syndrome includes acromegaly, hypothyroidism, hirsutism-related endocrinopathies such polycystic ovary syndrome, minoxidil use, or other rare pseudoacromegaly conditions such pachydermatoperiostosis, Berardinelli-Seip, Sotos, or Weaver syndromes; therefore, these patients are likely to be referred to adult or pediatric endocrine clinics [3, 4].

In summary, we present a five-member three-generation family with Cantú syndrome due to a novel missense variant in *ABCC9* gene showing full penetrance, and two family members with non-functioning pituitary adenomas. We show their acromegaloid facial phenotype over a 20-year-period combined with marked generalized hypertrichosis, and draw attention to their cardiac complications. This family also shows familial pituitary adenoma and, as this was not described in other patients with Cantú syndrome, it is unclear whether this feature is part of Cantú syndrome or a coincidental finding. Familial pituitary adenomas have a heterogeneous genetic background [14], and further studies are needed to see if there is indeed a link with *ABCC9*.
